# MELODI Presto: a fast and agile tool to explore semantic triples derived from biomedical literature

**DOI:** 10.1093/bioinformatics/btaa726

**Published:** 2020-08-18

**Authors:** Benjamin Elsworth, Tom R Gaunt

**Affiliations:** MRC Integrative Epidemiology Unit, Bristol Medical School, University of Bristol, Bristol BS8 2BN, UK; MRC Integrative Epidemiology Unit, Bristol Medical School, University of Bristol, Bristol BS8 2BN, UK

## Abstract

**Summary:**

The field of literature-based discovery is growing in step with the volume of literature being produced. From modern natural language processing algorithms to high quality entity tagging, the methods and their impact are developing rapidly. One annotation object that arises from these approaches, the subject–predicate–object triple, is proving to be very useful in representing knowledge. We have implemented efficient search methods and an application programming interface, to create fast and convenient functions to utilize triples extracted from the biomedical literature by SemMedDB. By refining these data, we have identified a set of triples that focus on the mechanistic aspects of the literature, and provide simple methods to explore both enriched triples from single queries, and overlapping triples across two query lists.

**Availability and Implementation:**

https://melodi-presto.mrcieu.ac.uk/.

**Supplementary information:**

Supplementary data are available at *Bioinformatics* online.

## 1 Introduction

The biomedical literature contains a wealth of knowledge on disease mechanisms that link risk factors to disease outcomes. Literature reviews are an important part of developing new mechanistic hypotheses, but are time-consuming when exploring many risk factors or disease outcomes in parallel. We propose utilizing the entire biomedical literature corpus to generate a metric of evidence based on the number of times a particular statement has been documented. This approach can be used to produce an overview of the main topics and terms for any given biomedical query, e.g. a risk factor, or identify overlapping elements between two queries, e.g. a risk factor (exposure) and disease outcome.

Previously, we created MELODI, a web application that derives overlapping enriched literature elements connecting a risk factor and a disease (Elsworth *et al.*, 2018), thus, identifying potential intermediate mechanisms. MELODI utilizes semantic triples (‘subject-predicate-object’) derived from the titles and abstracts of nearly 30 million biomedical articles using SemRep (Rindflesch and Fiszman, 2003) and provided by SemMedDB (Kilicoglu *et al.*, 2012). Whilst effective, the scale and complexity of data utilized by MELODI, its implementation of a graph database and web application, and its focus on single risk factor/outcome combinations limits its application to many queries in parallel.

To address these challenges, we developed MELODI Presto, a quicker and more agile tool to identify overlapping elements between any two query lists using millions of semantic triples from the scientific literature (https://melodi-presto.mrcieu.ac.uk/).

## 2 Features

The main features and innovations in MELODI Presto are described below:

Filter by UMLS semantic type: SemMedDB triples were filtered to include only those matching particular ‘term types’. These types are defined by the UMLS semantic type abbreviations (https://metamap.nlm.nih.gov/SemanticTypesAndGroups.shtml). We focus on terms most relevant to mechanistic inference. Supplementary Table S1 lists selected terms. For a triple to be included both the subject and object semantic types needed to be in this list.

Filter by predicate type: To improve the interpretability of the data, we removed the more ambiguous predicates. Supplementary Table S2 lists excluded predicates. We retained predicates that implied direction or causality, excluding predicates, such as PROCESS_OF, PART_OF and ISA. Combined, these two filtering criteria reduced the number of PREDICATION triples from 103 284 300 to 8 295 443.

Improve search performance: MELODI Presto implements a simpler approach than MELODI, which does not require a graph database architecture. We therefore selected Elasticsearch (https://www.elastic.co/elasticsearch) for performance based on our previous experience applying it to large biomedical datasets (Elsworth *et al.*, 2020).

## 3 Implementation

### 3.1 Data sources and pre-processing

MELODI Presto incorporates semantic triples from the SemMedDB database (Kilicoglu *et al.*, 2012), which is built by running the SemRep semantic knowledge representation tool (Rindflesch and Fiszman, 2003) on the MEDLINE database.

The SemMedDB resource (version semmedVER42_R, 2020) is updated periodically and provides data downloads in SQL format. For MELODI Presto, we extracted the PREDICATION, SENTENCE and CITATION tables. For enrichment analysis, frequency counts of triples were pre-calculated using Elasticsearch aggregation calls and added to a separate index. This will be updated with new SemMedDB releases.

The quick brown fox jumps over the lazy dog. The quick brown fox jumps over the lazy dog.

### 3.2 Functions

**Fig. 1.**
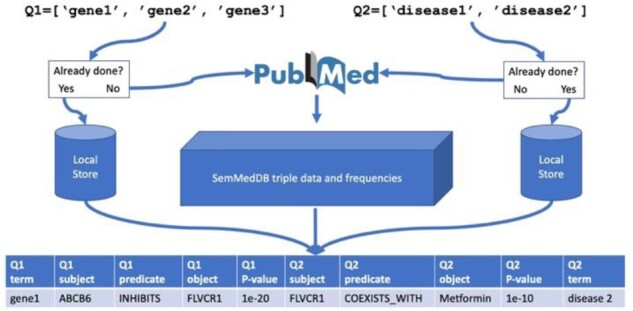
Data flow of the MELODI Presto overlap function. Two lists of queries (Q1 and Q2) are first checked for previous enrichment analysis. For queries, which have not been previously analysed the text of each missing term is run as a PubMed query and returned IDs are matched to the MELODI Presto database for enrichment. The results of previous analysis are loaded from a local store. Overlapping elements between each pair of enriched triple sets (one from each query list, Q1 and Q2) are then identified and returned

MELODI Presto provides three functions:

1. Enrich: The enrichment method follows the same principle as MELODI, using a standard 2 × 2 Fisher’s exact test. For example, if a query ‘Sleep duration’ returned a set of triples ‘Sleep Apnea, Obstructive: PREDISPOSES: Hypertensive disease’, then, we can count the number of this specific triple returned by the query (localCount), the total number of triples returned by the query (localTotal), the number of this specific triple in the database (globalCount) and the total number of triples in the database (globalTotal). These values are then provided as a 2 × 2 contingency table using a two-sided alternative hypothesis, producing a prior odds ratio and *P*-value (a=localCount, b=localTotal-localCount, c=globalCount, d=globalTotal-globalCount).
2. Overlap: This is an extension of the *Enrich* function described above. By providing two lists of search terms, e.g. multiple risk factors and multiple diseases, all terms are first tested for enrichment, then, overlapping enriched elements are identified. An overlap is taken to be cases where the object of a triple from the set of ‘x’ queries overlaps with a subject from the set of ‘y’ queries (Fig. 1).
3. Sentence: This function enables the user to check the source literature for SemMedDB triples. To enable rapid evaluation of the literature underpinning any potential mechanism, MELODI Presto provides a function that takes a PubMed ID and returns triples from the *refined* PREDICATE data, and the sentences from which they were derived.

### 3.3 Performance

The first time a query is run MELODI Presto creates local copies of the enrichment data, as illustrated above. For this reason, if a query has not been run previously, it may take a few seconds (generally <30 s depending on the number of articles returned from the query). However, if an existing variable, or list of variables, are queried, the *Overlap* function generally runs in a few seconds.

### 3.4 Access and source code

MELODI Presto is available via an application programming interface [API, using either the Swagger interface or an appropriate interface library (e.g. Python requests)]. Python examples of which can be found in the Jupyter notebooks linked below. Each function returns JSON objects, which can be easily incorporated into standard workflows. We also provide a web application to enable some of the functionality to be explored.

All code used to process the raw data, create the Elasticsearch indexes, API and web application are publicly available (https://github.com/MRCIEU/MELODI-Presto) with Jupyter notebooks providing a demonstration of basic API usage, use cases and details of specific methods and performance.

### 3.5 URLs

MELODI Presto—https://melodi-presto.mrcieu.ac.uk/.

MELODI Presto Web—https://melodi-presto.mrcieu.ac.uk/app/.

MELODI Presto API—https://melodi-presto.mrcieu.ac.uk/docs/.

MELODI Presto GitHub—https://github.com/MRCIEU/MELODI-Presto.

MELODI Presto Notebooks—https://github.com/MRCIEU/MELODI-Presto/blob/master/notebooks/.

## 4 Conclusion

MELODI Presto provides a fast and efficient method to systematically profile semantic triples derived from the literature. This can be used to explore the enriched literature data for a given search term and identify potential intermediate disease mechanisms between lists of terms. Using a refined literature dataset and a high-performance search architecture, it is possible to query millions of articles in seconds.

The agility of the method and its construction mean that as and when alternative or improved triples of data are produced, we can add them (Koroleva *et al.*, 2020; Sybrandt *et al.*, 2020). There is also the scope to expand this approach to include preprints, full text and any other source of data.

## Funding

This work was supported by the UK Medical Research Council Integrative Epidemiology Unit [MC_UU_00011/4]; the Cancer Research UK Integrative Cancer Epidemiology Programme [C18281/A19169]; and the University of Bristol. T.R.G. holds a fellowship from the Alan Turing Institute. T.R.G. receives funding from GlaxoSmithKline and Biogen for unrelated research.

## Supplementary Material

btaa726_Supplementary_Data
