# Supplementary material for: MELODI Presto: a fast and agile tool to explore semantic triples derived from biomedical literature
Source: Bioinformatics. 2020 Aug 18;37(4):583–5. doi: 10.1093/bioinformatics/btaa726 (PMC8088324; doi:10.1093/bioinformatics/btaa726)
Supplement: btaa726_Supplementary_Data [file btaa726_supplementary_data.docx]

# SUPPLEMENTARY DATA

# MELODI Presto: A fast and agile tool to explore semantic triples derived from biomedical literature

Benjamin Elsworth and Tom R Gaunt

Supplementary table 1. UMLS semantic types and their counts **included** in MELODI Presto.

| Type | Type full name | Subject Count | Object Count |
| --- | --- | --- | --- |
| aapp | Amino Acid, Peptide, or Protein | 2,999,120 | 1,622,503 |
| gngm | Gene or Genome | 1,296,739 | 2,148,741 |
| orch | Organic Chemical | 1,267,784 | 651,827 |
| phsu | Pharmacological Substance | 1,135,742 | 290,272 |
| dsyn | Disease or Syndrome | 935,464 | 3,063,668 |
| horm | Hormone | 253,615 | 120,118 |
| hops | Hazardous or Poisonous Substance | 183,193 | 111,964 |
| inch | Inorganic Chemical | 148,322 | 176,647 |
| enzy | Enzyme | 39,203 | 50,128 |
| chem | Chemical | 17,092 | 14,860 |
| clnd | Clinical Drug | 10,747 | 1,043 |
| clna | Clinical Attribute | 8,422 | 43,672 |

Supplementary table 2. SemMedDB predicates and counts **excluded** from MELODI Presto

| Predicate | Triple Count |
| --- | --- |
| PROCESS_OF | 20,972,485 |
| LOCATION_OF | 17,480,766 |
| PART_OF | 10,291,675 |
| ISA | 6,204,528 |
| USES | 4,698,948 |
| ADMINISTERED_TO | 1,616,069 |
| compared_with | 1,113,762 |
| METHOD_OF | 607,727 |
